# Modern Disposable E‑Cigarettes: Small in Size but Big in Toxic Metals

**DOI:** 10.1021/acscentsci.5c01312

**Published:** 2025-08-05

**Authors:** Pablo Olmedo, Fernando Gil

**Affiliations:** † Department of Legal Medicine and Toxicology, School of Medicine, 16741 University of Granada, 18016 Granada, Spain; ‡ Instituto de Investigación Biosanitaria ibs.GRANADA, 18012 Granada, Spain

## Abstract

Safety concerns continue to arise as lead and other potentially toxic metals are found in the aerosols of modern disposable e-cigarettes.

## Graphical abstract

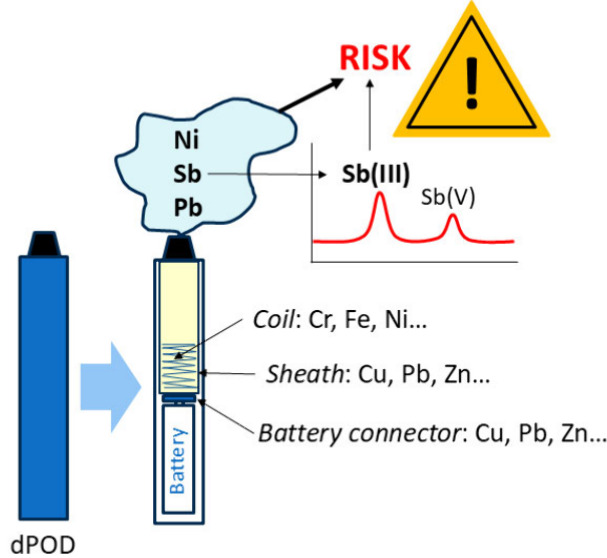

E-cigarettes are often regarded as a healthier alternative to conventional tobacco products. However, they are a source of a wide variety of potentially toxic substances. Among these toxicants, heavy metals such as lead have been identified in the aerosol generated in e-cigarettes. These metals might come from the components within these devices that are heated to generate the aerosol.^1^ Each device contains a metallic coil that heats a liquid (also called e-liquid), producing an aerosol (vapor) that is inhaled by the user (vaper).

Interestingly, e-cigarettes have evolved considerably over the years. A decade ago, tank-style e-cigarettes (usually referred to as MODs) were the most popular. MODs are rechargeable e-cigarettes with a refillable tank for the e-liquid. They frequently have powerful batteries that can generate massive amounts of aerosol, and therefore, the metal transfer from the device to the aerosol can be intense.^1,2^ More recently, compact reusable e-cigarettes with changeable prefilled cartridges (also called PODs) became the most popular. These devices also release metals.^3^

Nowadays, small disposable devices (also called dPODs) are the most popular e-cigarettes, particularly among adolescent children.^4^

Although their appearance is not as impressive as the big and powerful tank-style e-cigarettes, these newer devices might share the same risks to human health.

In this issue of *ACS Central Science*, Poulin and co-workers investigated the exposure to metals from these modern devices.^5^ In addition to the analysis of metals in the aerosol of three popular brands of dPODs, this study also focused on examining the composition of the heating coils and other metallic parts of the e-cigarettes, which can contribute to metal emissions. Furthermore, metal concentrations were determined in the e-liquids before and after their use, and different compositions of flavorings in the e-liquids were also studied. An important strength of this study compared to others is the determination of the oxidation states of certain elements. The oxidation state of elements such as chromium and antimony can have a significant influence on their toxicity: hexavalent chromium Cr­(VI) and trivalent antimony Sb­(III) are carcinogenic, while trivalent chromium Cr­(III) and pentavalent antimony Sb­(V) are not.^6,7^

Poulin and colleagues^5^ found that the heating coils contained metals such as chromium (Cr), iron (Fe), and nickel (Ni), while nonheating parts (sheaths and battery connectors) showed a different composition, including metals such as copper (Cu), lead (Pb), and zinc (Zn). It is concerning that some parts of one of the devices showed high proportions of Pb, as it is an extremely toxic metal that should not be used in the manufacturing of e-cigarettes (Figure 1). These nonheating components can contaminate the e-liquid with these metals even before using the e-cigarette. On the other hand, metals from the heating coil (mainly Cr and Ni) were transferred to the aerosol in larger amounts as the e-cigarettes were used. It is also suggested that flavorings can play a role in the transfer of these metals from the heating coil. The contamination with metal coming from the heating coil was confirmed by analyzing the e-liquid after the e-cigarette was used. Indeed, the used e-liquids contained remarkably higher concentrations of Cr and Ni than the unused e-liquids. Other elements such as Pb, Cu, Zn, and Sb also showed high concentrations in aerosols and used e-liquids.

**Figure 1:**
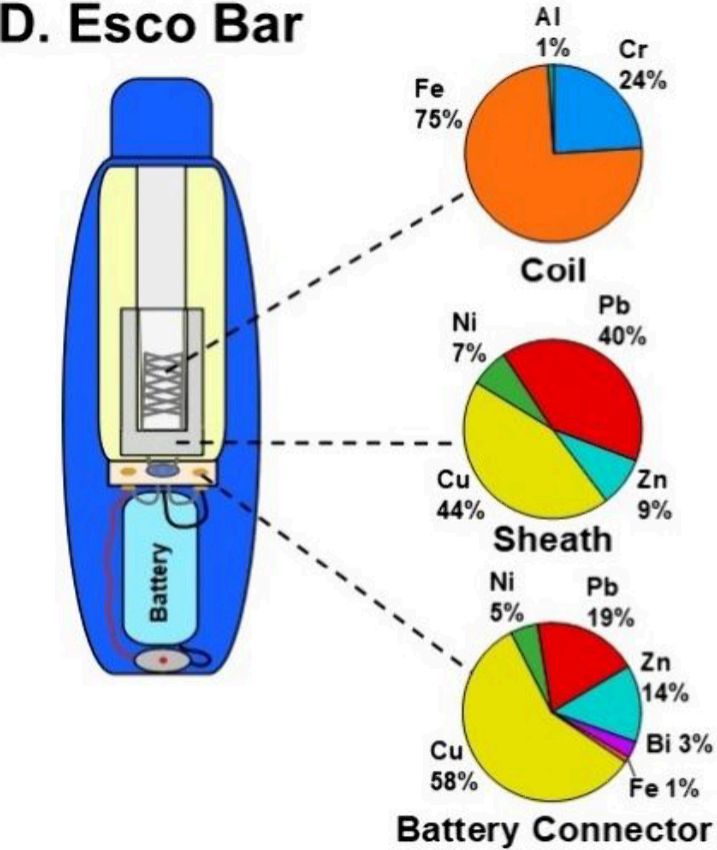
Elemental compositions of device coil, sheath, and battery connector of disposable e-cigarette (dPOD) Esco Bar. Reproduced from ref 5. Available under a CC-BY 4.0 license. Copyright 2025 Mark R. Salazar, Lalima Saini, Tran B. Nguyen, Kent E. Pinkerton, Amy K. Madl, Austin M. Cole, and Brett A. Poulin.

Unfortunately, these metals were also found in the aerosol of earlier generations of e-cigarettes.^8^ Over the past decade, many studies have reported high levels of metals such as Ni, Cr, Pb, Cu, and Zn in the e-cigarette aerosol and the e-liquid after the device was used.^1–3,9^ However, the study by Poulin and colleagues provides a deeper perspective on the origin of these metals.^5^ Each part of the e-cigarette contributes to the exposure to different metals.

Also, unacceptable materials such as leaded bronze alloys used to manufacture some of these devices are responsible for the particularly concerning exposure to lead.

In fact, the emission of lead from these devices is higher than that from conventional tobacco cigarettes. Therefore, this investigation opens the possibility of conducting further research on the materials used to make these devices, detecting potentially unsafe components, and proposing alternative materials and designs that could minimize the exposure to toxicants through e-cigarettes.

Another relevant aspect of this study is the oxidation states of some elements found in the e-cigarette aerosol. While carcinogenic Cr­(VI) was not detected in the aerosol, a notable percentage of the antimony was carcinogenic Sb­(III) in the aerosol of some devices.^5^

Furthermore, Ni, Sb­(III), and Pb data were used to assess the cancer and noncancer risk in daily users, finding that the exposures to these elements exceeded the limits considered safe. This is especially concerning in the case of lead exposure in children, as they are more susceptible to the neurological damage produced by this metal.

In view of the risks described in this investigation, these interesting results should encourage future research on this matter and further control measures to protect the health of consumers.^10^

In conclusion, Poulin and colleagues have provided more evidence that the use of e-cigarettes is not as safe as it might appear. Modern dPODs continue to be a source of exposure to metals, including extremely toxic lead, similar to devices of earlier generations. This study is brilliantly designed, conducted with advanced techniques, and provides solid data on the origin of metal contamination in e-cigarette aerosols inhaled by the users. Therefore, this article is extremely relevant to evaluate the current situation of e-cigarette safety and contains information of interest to consumers, manufacturers, and policymakers.
